# Self-assembled monolayers of shape-persistent macrocycles on graphite: interior design and conformational polymorphism

**DOI:** 10.3762/bjoc.10.294

**Published:** 2014-11-26

**Authors:** Joscha Vollmeyer, Friederike Eberhagen, Sigurd Höger, Stefan-S Jester

**Affiliations:** 1 Kekulé-Institut für Organische Chemie und Biochemie, Rheinische Friedrich-Wilhelms-Universität Bonn, Gerhard-Domagk-Str. 1, 53121 Bonn, Germany

**Keywords:** conformational polymorphism, self-assembled monolayers, shape-persistent macrocycles, solid/liquid interface, supramolecular surface patterning, template

## Abstract

Three shape-persistent naphthylene–phenylene–acetylene macrocycles of identical backbone structures and extraannular substitution patterns but different (empty, apolar, polar) nanopore fillings are self-assembled at the solid/liquid interface of highly oriented pyrolytic graphite and 1,2,4-trichlorobenzene. Submolecularly resolved images of the resulting two-dimensional (2D) crystalline monolayer patterns are obtained by in situ scanning tunneling microscopy. A concentration-dependent conformational polymorphism is found, and open and more dense packing motifs are observed. For all three compounds alike lattice parameters are found, therefore the intermolecular macrocycle distances are mainly determined by their size and symmetry. This is an excellent example that the graphite acts as a template for the macrocycle organization independent from their specific interior.

## Introduction

One of the ultimate aims in supramolecular chemistry on solid surfaces is the formation of two-dimensional (2D) nanostructures that are capable of performing highly specific tasks as an effect of functional units that are implemented into the adlayer building blocks. An efficient functionality of such architectures will essentially rely on a precise control of the orientation and distribution of the molecules on the surface that is summarized in the field of 2D supramolecular engineering [1–2]. Shape-persistent arylene–alkynylene macrocycles are promising candidates as future mounts for functional units. These are separated from each other and cannot interact intermolecularly if the rings are adsorbed in parallel to the substrate. The exterior of the macrocycles can be substituted with alkyl side chains (or their alkoxy analogs) that guarantee a sufficient compound solubility. The macrocycles can be co-assembled with other compounds at the surface [3–4], and they can also template the subsequent organization of guest molecules and thus a growth into the third dimension [5–6]. One of the most often used substrates for supramolecular surface patterning is highly oriented pyrolytic graphite (HOPG) which provides large atomically flat terraces between step edges and a sufficient adsorbate mobility, required for the self-assembly process that leads to the 2D crystal formation.

For a more detailed understanding of the macrocycle–HOPG and macrocycle–macrocycle interactions, the following key aspects must be considered:

(i) how the extraannular alkyl side chains of the adsorbed macrocycles pack, and whether this can be compared to the packing of linear hydrocarbons on HOPG,

(ii) how the specific attachment of the extraannular alkyl chains at the macrocycle rims affects the packing, and

(iii) how the ring interior influences the packing of the macrocycles on HOPG.

**Figure 1:**
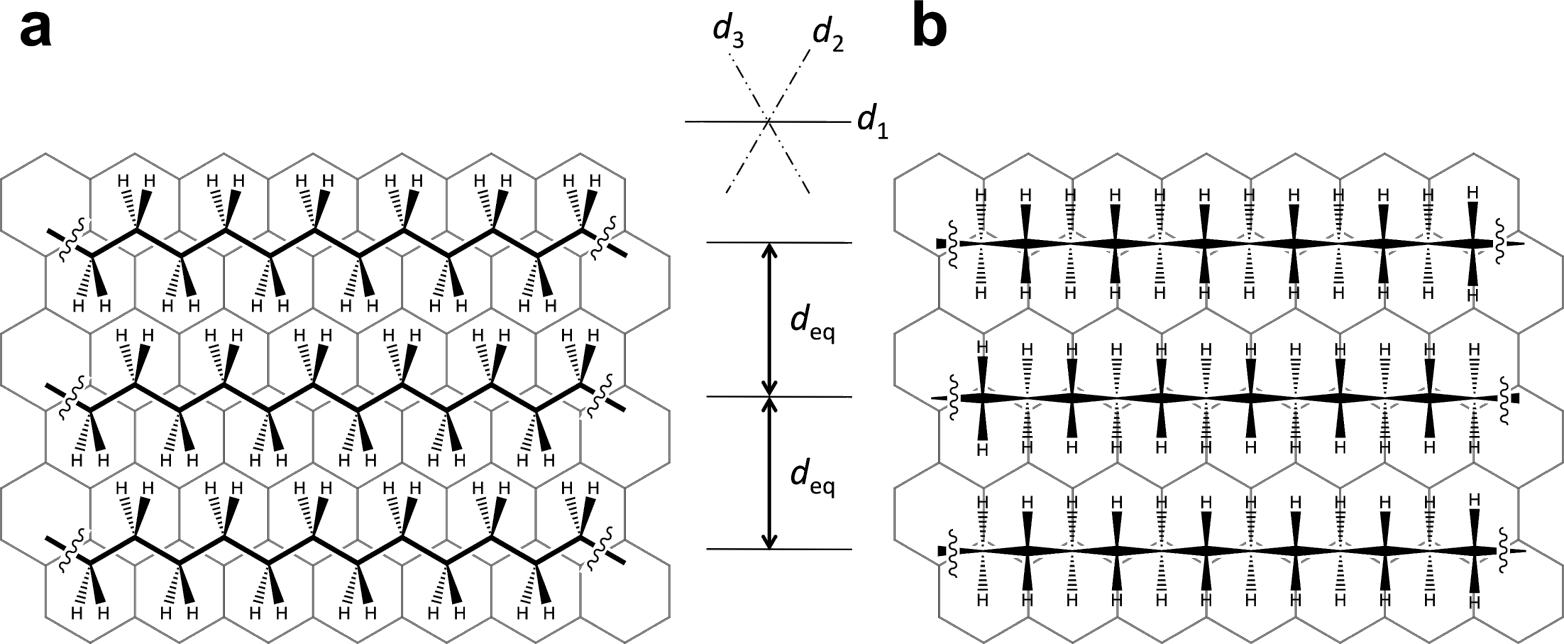
(a), (b) Two distinguishable packings of alkyl chains on cutouts of graphite are shown with the carbon backbones aligned (a) coplanar with the graphite surface [7–12], and (b) perpendicular to the graphite surface [9–12]. In both cases the chains align along one of the substrate main axis directions (i.e., *d*_1_, *d*_2_, or *d*_3_) and adopt equilibrium distances of (a) *d*_eq_ = 0.43 nm or (b) *d*_eq_ = 0.35 nm [10].

First we address the structure of self-assembled alkyl chains on HOPG. Their methylene units adopt a staggered (anti) conformation and align along one of the three crystallographic main axis directions of the substrate. Their carbon backbones either orient (as most often observed) coplanar with the graphite surface (Figure 1a) and adopt equilibrium interchain distances, *d*_eq_, of 0.43 nm [7–12], or they orient in an (also reported) stacked fashion (with the carbon backbones axially rotated by 90°; Figure 1b) and a *d*_eq_ of 0.35 nm [9–12].

**Figure 2:**
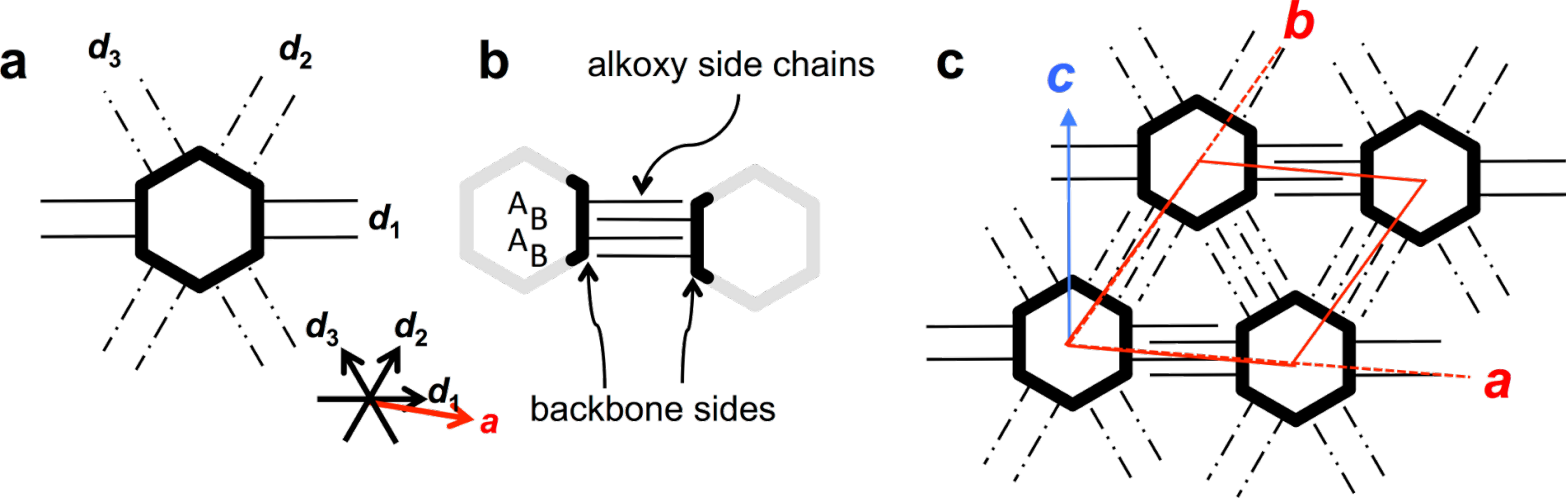
(a)–(c) Schematic structure of a hexagonal shape-persistent macrocycle with extraannular alkoxy chains at its sides, the side chain interaction scheme, and the resulting pattern geometry. (a) Two (flexible, linear) alkoxy side chains of each hexagon side point along the normals of the latter. (b) Two side chains of neighboring hexagon sides interdigitate in an ABAB binding motif. Identical van der Waals binding occurs along all six directions determined by the three crystallographic main axes *d*_1_, *d*_2_, and *d*_3_. (c) Hexagonal pattern of molecular hexagons where all alkoxy side chains are adsorbed on the substrate along the six HOPG main axis directions, *d*_1_, *d*_2_, and *d*_3_, that are defined by the arrows in (a). Backbones are shown as bold black and grey lines, and the alkoxy side chains that after adsorption align along *d*_1_, *d*_2_, and *d*_3_ are shown as solid lines, dash-dotted lines, and dash-double-dotted lines, respectively. The red lines indicate unit cell vectors *a* and *b*; the blue arrow indicates the backbone orientation, *c*.

Shape-persistent macrocycles on HOPG form non-covalent 2D assemblies held together by the van der Waals interaction between the alkyl side chains even without the presence of any additional functional groups [13–15]. The templated alignment of the alkyl chains on the surface determines the commensurability or registry of the adsorbate vs substrate lattice vectors – even if extended rigid backbones act as cores of certain sizes and shapes [16] that provide anchor units for the alkyl chains. However, often the 2D superstructures are not predictable [17] or show a conformational polymorphism [18], also as an effect of varying compound concentrations in the supernatant solution [19–20]. A recent approach investigating macrocycles of triangular, quadratic, pentagonal, and hexagonal shapes that carry alkoxy side chains pointing away in normal direction from their sides has led to the concept of molecular polygons [4]. An example for the schematic design of an alkoxy side chain substituted molecular hexagon and its characteristic side-chain interdigitation concept are shown in Figure 2a and b, respectively. Two alkoxy side chains of each side interdigitate with two side chains of an adjacent macrocycle and form an ABAB interdigitation pattern along each HOPG main axis direction.

An increase of the concentration of the adsorbate molecules in the supernatant solution leads generally to denser but often significantly less specific or amorphous packing morphologies, as an uncharacteristic but variable number of the side chains are no longer adsorbed on the substrate, but point towards the solution phase [4]. The molecule–molecule interaction strength and the intermolecular distances originate from the length and the packing of the side chains that are adsorbed on the substrate. Reducing the symmetry of the hexagon, or reducing the numbers of side chains on some of the hexagon sides, should lead to an unequal van der Waals interaction strength along different directions, which would consequently allow also a prediction of the surface pattern at high concentrations. This should lead to a tailorable structure of both, the porous and dense polymorphs, or – in other words – an alteration between two discrete designable packings, here as an effect of the compound concentration in the supernatant solution phase. Note that contrary to “alterable” packings, the term “adaptable” has been previously applied to indicate (side chain substituted) units in shape-persistent macrocycles that change their orientation with respect to the overall backbone, e.g., as an effect of solvophobic effects by a rotation of the corresponding *p*-phenylenes [21].

In general, in order to achieve predictable 2D adsorbate geometries of shape-persistent macrocycles, as adjustable with atomic scale definition, it is essential that the unit cell parameters (and the packing architectures in general) do not change when the central unit is varied. The driving forces for their 2D self-assembly are most probably independent from the presence of additional functional groups pointing into the third dimension or located inside the cavity interior. To the best of our knowledge, only little effort has been spent so far on investigating the role of intraannular substituents on the 2D supramolecular surface patterns of macrocyclic compounds. Therefore, in addition to the concentration-driven conformational polymorphism that is yet attributed to a distinct extraannular substitution pattern as discussed above, we evaluate the role of the intraannular substitution on the 2D supramolecular self-assembly of macrocycles.

**Figure 3:**
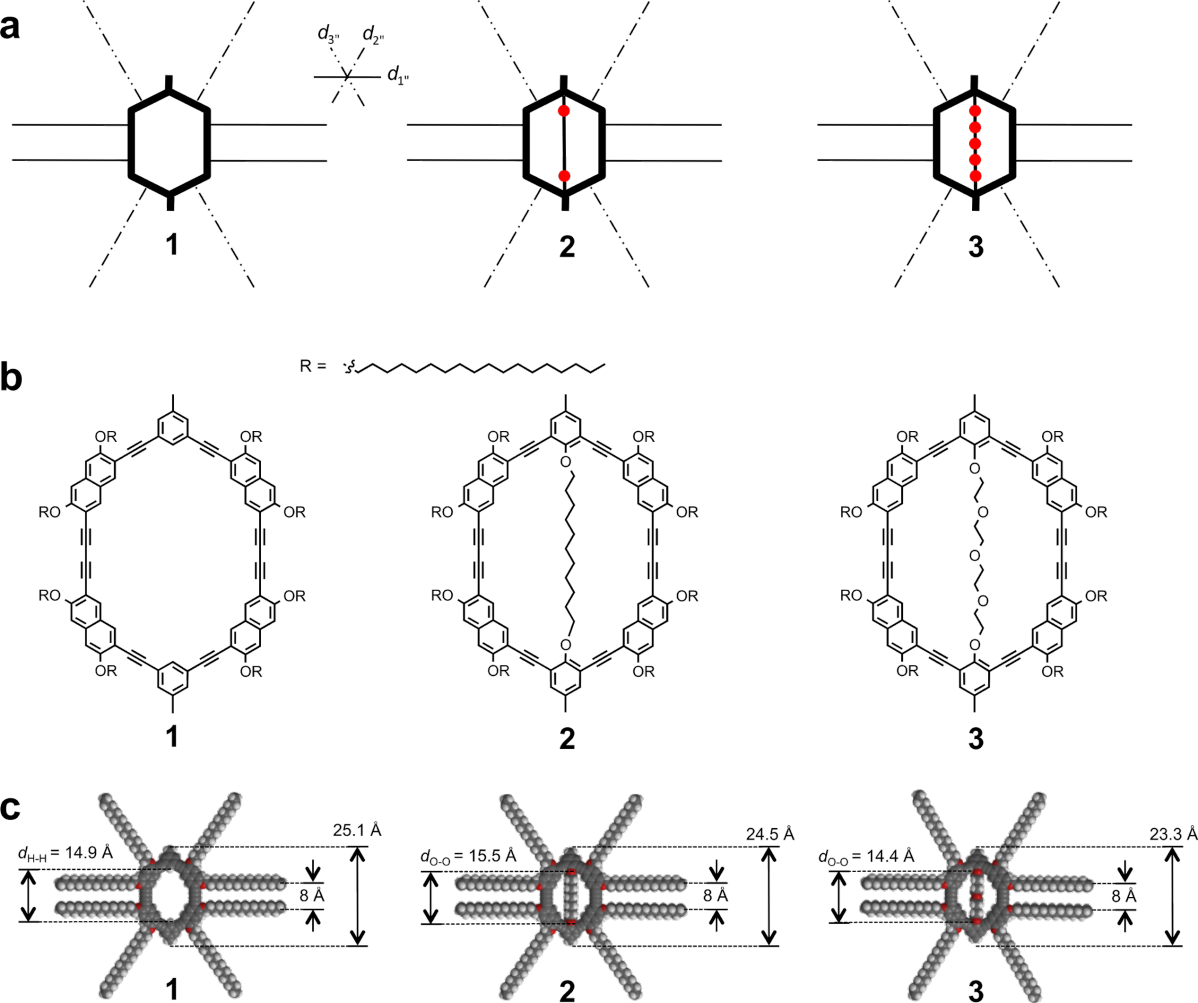
(a)–(c) Shape-persistent macrocycles with an empty cavity (**1**), an apolar interior (undecyl diether strand, **2**), and a polar interior (tetraethylene glycol strand, **3**). (a) Schematic structures. The bold lines represent the (identical) macrocycle backbones; the thin straight, dash-dotted, and dash-double-dotted lines indicate alkoxy side chains. (b) Chemical structures. (c) Molecular models. Backbone geometries of the shape-persistent macrocycles with different interiors were derived by force-field modelling (Spartan ‘08) restricted to 2D (including interaction with a graphene layer with fixed atom positions), and extraannular octadecyloxy side chains in staggered (anti) conformation were subsequently added (along *d*_1_, *d*_2_, and *d*_3_ directions to adopt 60°/120° angles). The macrocycle sizes are indicated, and the cavity sizes *d*_H-H_ for **1** and *d*_O-O_ for **2** (with the 1,11-undecanediol interior) as well as for **3** (with the tetraethylene glycol interior) are given. Note that slightly varying macrocycle sizes are induced by the intraannular strands and vary by less than 2 Å. (b) and (c) are adapted with permission from [22]. Copyright 2012 The Royal Society of Chemistry.

Compounds **1**–**3** (Figure 3) have the same macrocyclic rigid backbone and flexible octadecyloxy periphery and differ only in their intraannular substitution. While **1** has an empty interior, **2** contains an alkyl chain crossing the ring, and **3** a polar oligoether chain. The macrocycles are composed of four naphthylene units as upper and lower east and west corner building blocks, whereas the north and south corners are phenylene units.

## Results and Discussion

**Figure 4:**
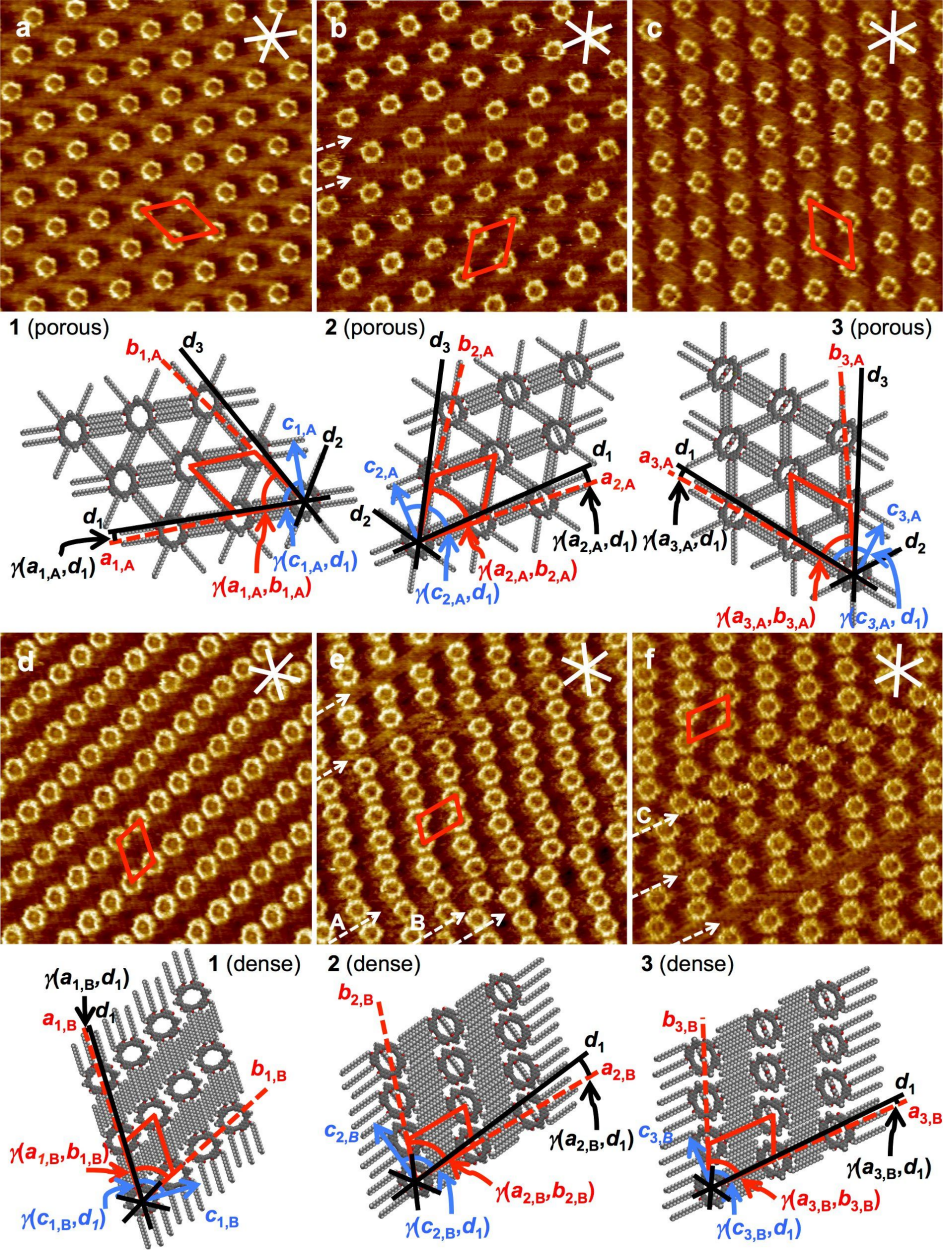
Scanning tunneling microscopy images and supramolecular models of (a)–(c) porous (= polymorph A) and (d)–(f) dense patterns (= polymorph B) of **1**–**3** at the TCB/HOPG interface. Image parameters, unit cells, and additional packing parameters are for the porous patterns: (a) **1**; *c* = 2 × 10^−6^ M, *V*_S_ = −1.0 V, *I*_t_ = 12 pA; *a*_1,A_ = 4.6 ± 0.2 nm, *b*_1,A_ = 4.7 ± 0.2 nm, γ(*a*_1,A_,*b*_1,A_) = 57 ± 2°; γ(*a*_1,A_,*d*_1_) = 3 ± 2°; γ(*c*_1,A_,*d*_1_) = 90 ± 3°; (b) **2**; *c* = 2 × 10^−6^ M, *V*_S_ = −1.0 V, *I*_t_ = 7 pA; *a*_2,A_ = 4.6 ± 0.2 nm, *b*_2,A_ = 4.7 ± 0.2 nm, γ(*a*_2,A_,*b*_2,A_) = 57 ± 2°; γ(*a*_2,A_,*d*_1_) = 5 ± 2°; γ(*c*_2,A_,*d*_1_) = 90 ± 3°; (c) **3**; *c* = 3 × 10^−6^ M, *V*_S_ = −1.2 V, *I*_t_ = 15 pA; *a*_3,A_ = 4.6 ± 0.2 nm, *b*_3,A_ = 4.7 ± 0.2 nm, γ(*a*_3,A_,*b*_3,A_) = 57 ± 2°; γ(*a*_3,A_,*d*_1_) = 3 ± 2°; γ(*c*_3,A_,*d*_1_) = 90 ± 3°; and for the dense patterns: (d) **1**; *c* = 10^−5^ M, *V*_S_ = −1.2 V, *I*_t_ = 5 pA; *a*_1,B_ = 4.6 ± 0.2 nm, *b*_1,B_ = 2.9 ± 0.2 nm, γ(*a*_1,B_,*b*_1,B_) = 66 ± 2°; γ(*a*_1,B_,*d*_1_) = 1 ± 2°; γ(*c*_1,B_,*d*_1_) = 90 ± 3°; (e) **2**; *c* = 10^−5^ M, *V*_S_ = −1.2 V, *I*_t_ = 30 pA; *a*_2,B_ = 4.4 ± 0.2 nm, *b*_2,B_ = 2.7 ± 0.2 nm, γ(*a*_2,B_,*b*_2,B_) = 71 ± 2°; γ(*a*_2,B_,*d*_1_) = 6 ± 2°; γ(*c*_2,B_,*d*_1_) = 90 ± 3°; (f) **3**; *c* = 10^−5^ M, *V*_S_ = −1.2 V, *I*_t_ = 10 pA; *a*_3,B_ = 4.6 ± 0.2 nm, *b*_3,B_ = 3.1 ± 0.2 nm, γ(*a*_3,B_,*b*_3,B_) = 68 ± 2°; γ(*a*_3,B_,*d*_1_) = 2 ± 2°; γ(*c*_3,B_,*d*_1_) = 90 ± 3°). All image sizes are 32.7 × 32.7 nm^2^. The red lines indicate the unit cells, *a_n_*_,_*_m_*, *b_n_*_,_*_m_*, γ(*a_n_*_,_*_m_*,*b_n_*_,_*_m_*), the white and black lines indicate the HOPG main axis directions, *d*_1_, *d*_2_, *d*_3_, and the blue arrows indicate the north–south axis directions of the backbones, *c_n_*_,_*_m_*, *n* = 1, 2, 3; *m* = A, B. The dashed white arrows in (b), (e), and (f) point out packing faults. In particular, arrows A–C point out packing faults where the lines of macrocycles are shifted along the HOPG main axis direction *d*_1_ by the length of one or more –CH_2_– units. The STM image in (e) is adapted with permission from [22]. Copyright 2012 The Royal Society of Chemistry.

All three compounds **1**–**3** form porous (“low concentration”, Figure 4a–c) and dense (“high concentration”, Figure 4d–f) adsorbate patterns, depend on the compound concentrations, as observed by scanning tunneling microscopy (STM).

**Figure 5:**
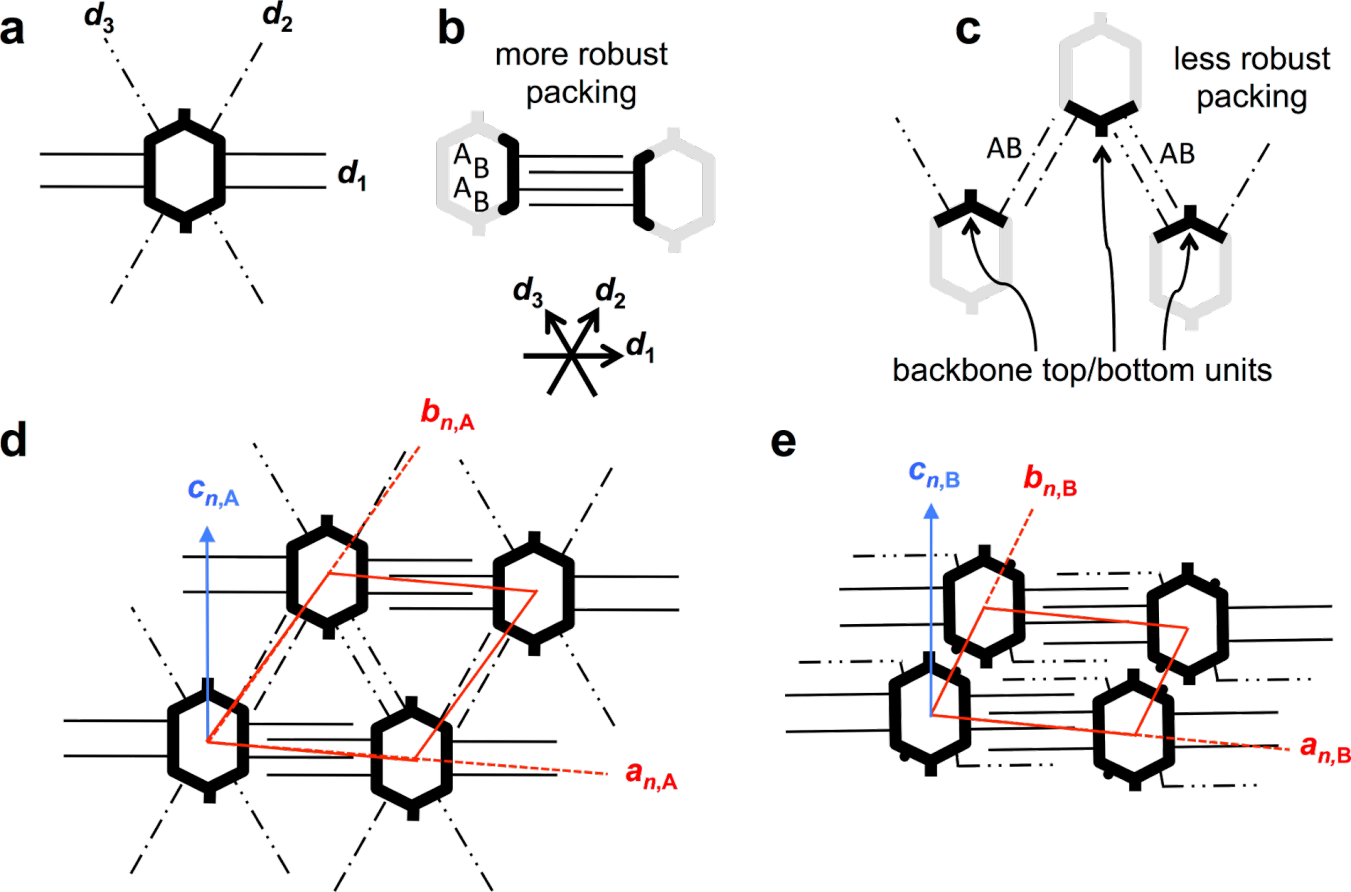
(a)–(e): Schematic structure of the shape-persistent macrocycle **1** (as a representative of the series of **1**–**3** that form the same patterns), its alterable side chain interaction schemes, and the resulting pattern geometries. (a) Eight alkoxy side chains are attached to four corners of the backbone. After adsorption, four side chains align along the crystallographic main axis direction *d*_1_, and four alkoxy side chains align along directions *d*_2_ and *d*_3_. (b) Two alkoxy side chains of neighboring sides form an ABAB interdigitation motif along *d*_1_ (more robust packing). (c) Two side chains of neighboring macrocycles align along directions *d*_2_, and *d*_3_, here denoted as AB packing motif (less robust packings). (d) Porous pattern (polymorph A) of macrocycles where all alkoxy side chains are adsorbed on the substrate. (e) Oblique packing (polymorph B) of the molecules where six of the eight alkoxy side chains (black solid and dash-double-dotted thin lines) of each molecule are adsorbed on the substrate, whereas two side chains of each molecule point towards the solution phase (black dots). The arrows in (b) define the HOPG crystallographic main axis directions *d*_1_, *d*_2_, and *d*_3_. The backbones are shown as bold black and grey lines with alkoxy side chains that after adsorption align along *d*_1_ (thin solid lines), *d*_2_ (thin dash-dotted lines), and *d*_3_ (thin dash-double-dotted lines). The red lines indicate the unit cell vectors *a_n_*_,m_ and *b_n_*_,m_; the blue arrows indicate the macrocycle north–south axis directions, *c_n_*_,m_.

Bright and dark parts in the STM images correspond to regions covered by aromatic backbones and alkoxy side chains, respectively [23], whereas the medium bright image color mostly represents regions covered by solvent molecules. For the porous patterns (polymorph A) of all three compounds, unit cells of *a_n_*_,A_ = 4.6 ± 0.2 nm, *b_n_*_,A_ = 4.7 ± 0.2 nm, γ(*a_n_*_,A_,*b_n_*_,A_) = 57 ± 2°, *n* = 1, 2, 3, are indexed and are undistinguishable within the experimental resolution. The orientations of the backbones are defined by their north–south-axis directions *c_n_*_,A_, *n* = 1, 2, 3, and all backbones are oriented with γ(*c_n_*_,A_,*d*_1_) = 90 ± 3° relative to the HOPG main axis direction *d*_1_. In addition, the alignment of the unit cell vectors *b_n_*_,A_, *n* = 1, 2, 3, with respect to *d*_1_ is γ(*a*_1,A_,*d*_1_) = 3 ± 2°, γ(*a*_2,A_,*d*_1_) = 5 ± 2°, and γ(*a*_3,A_,*d*_1_) = 3 ± 2°, and the values do not vary within the experimental error. In other words, the packing of all three compounds is independent on whether the cavity is empty (**1**), filled by an undecyl diether (**2**), or a tetraethylene glycol diether (**3**) strand. Rather, it is a result of the chemical structures of the backbones and extraannular side chains that is schematically represented in Figure 5a. Each naphthylene unit is 2,7-disubstituted to realize the 120° angle in the rigid macrocyclic backbone (Figure 3b), and carries additional octadecyloxy side chains at positions 3 and 6 of which one points along *d*_1_ in the porous pattern (cf. Figure 5a). They form an ABAB packing motif (along *d*_1_, cf. Figure 5b) and are (mostly) resolved by STM (Figure 4a–c). The other four alkoxy side chains of the four naphthylene corners point towards each of the four directions along *d*_2_ and *d*_3_ (cf. Figure 5a), with γ(*d*_1_,*d*_2_) = γ(*d*_2_,*d*_3_) = 60°/120° as defined by the HOPG substrate, and form AB alignment motifs (Figure 5c). Although the latter remain unresolvable by STM, the observed packing can only be explained by the alkoxy side chains oriented in this fashion [7–12].

If the compound concentrations (of **1**–**3** in TCB, respectively) are increased (from 2–3 × 10^−6^ M to 10^−5^ M), denser packings are observed (polymorph B) as shown in Figure 4d–f. The indexed unit cells are for compound **1**: *a*_1,B_ = 4.6 ± 0.2 nm, *b*_1,B_ = 2.9 ± 0.2 nm, γ(*a*_1,B_,*b*_1,B_) = 66 ± 2°, for compound **2**: *a*_2,B_ = 4.4 ± 0.2 nm, *b*_2,B_ = 2.7 ± 0.2 nm, γ(*a*_2,B_,*b*_2,B_) = 71 ± 2°, and for compound **3**: *a*_3,B_ = 4.6 ± 0.2 nm, *b*_3,B_ = 3.1 ± 0.2 nm, γ(*a*_3,B_,*b*_3,B_) = 68 ± 2°. This means that the unit cells vary slightly but significantly with respect to the experimental error for the three compounds. A further evaluation of the results requires a more detailed inspection of the packings. The alignment of the unit cell vectors *a_n_*_,B_, *n* = 1, 2, 3, with respect to *d* is γ(*a*_1,B_,*d*_1_) = 1 ± 2°, γ(*a*_2,B_,*d*_1_) = 6 ± 2°, and γ(*a*_3,B_,*d*_1_) = 2 ± 2° and thus identical for **1** and **3** within the experimental error, whereas it is slightly different for **2**. The orientation of the backbones *c_n_*_,B_, *n* = 1, 2, 3, is again γ(*c_n_*_,B_,*d*_1_) = 90 ± 3° relative to the HOPG main axis direction *d*_1_. Five side chains of two adjacent macrocycles are aligned along the crystallographic main axis direction *d*_1_ and form an ABABA interdigitation motif (Figure 5e) and give rise to a certain robustness of the latter. However, the binding motif of two side chains that align along directions *d*_2_ and *d*_3_ between each two macrocycles, here denoted as an AB packing motif (and observed in polymorph A, Figure 4a–c, and schematically shown in Figure 5c) is no longer found in polymorph B.

In other words, at higher concentration the packing changes compared to the low-concentration polymorph can be described as following:

(i) The alkoxy side chains indicated as solid lines in Figure 5d and e have the same alignment in both polymorphs (robust packing);

(ii) two of the side chains of each macrocycle that stabilize the less robust packing motifs (which are indicated as dash-double-dotted lines in Figure 3a as well as Figure 5d and e) alter between different alignment directions in both polymorphs, along *d*_3_ in the porous pattern in Figure 5d and along *d*_1_ in the dense pattern in Figure 5e; and

(iii) two side chains (which are indicated as dash-dotted lines in Figure 3a and Figure 5d) point towards the solution phase (and are represented as dots in Figure 5e).

Note that the alkyl chains that point towards to the solution phase may, in principle, interfere with the STM tip. However, the high flexibility, the low electric conductivity, and the low number of only two dangling alkyl chains per molecule allow a (rather) undisturbed STM imaging. In addition, the molecules are still fixed on the substrate by the remaining six adsorbed side chains and the dense packing.

The packing alters from the porous polymorph A, a highly symmetric hexagonal pattern of macrocycles (where all alkoxy side chains are adsorbed on the substrate which is similar to the pattern discussed for the molecular hexagons, Figure 2) to the dense polymorph B, an oblique packing. Thereby the coordination numbers of the molecules – defined as the directions along which the side chains stabilize the network [15] – are reduced from six in polymorph A (Figure 4a–c, Figure 5a–d) to two in polymorph B (Figure 4d–f, Figure 5e). Both supramolecular patterns are closely related to their backbone structures and substitution schemes.

While the lattice parameters of the porous packings (polymorph A) are identical for all compounds, **1**–**3**, the lattice parameters of the dense packings (polymorph B) vary slightly, depending on whether the cavity is empty (**1**), filled with an undecyl diether strand (**2**), or a tetraethylene glycol strand (**3**). In the porous polymorphs (of each compound), the alkoxy side chains maximize their overlap, and both lattice constants are a direct effect of the side chain lengths. In the dense polymorphs, the packing along lattice vector *a_n_*_,B_ (*n* = 1, 2, 3) is similarly a direct result of the side chain length. Contrary, the packing along the lattice vector *b_n_*_,B_ (*n* = 1, 2, 3) is a result of two effects:

(i) It results from the packing density of six interdigitating side chains, and the distance between each two neighboring chains is ca. 0.4 nm, as discussed above.

(ii) It is an effect of the steric requirement of the northeast and the southwest parts of neighboring macrocycle backbones. The backbone sizes of **1**–**3** vary slightly as an effect of the macrocycle interior, and are 25.1 Å for **1**, 24.5 Å for **2**, and 23.3 Å for **3** (cf. Figure 3c).

The angle γ(*a_n_*_,B_,*b_n_*_,B_) between the unit cell vectors *a* and *b* (for *n* = 1, 2, 3) is a result of the interlocking of the –CH_2_– units of alkoxy side chains neighboring macrocycles. The macrocycles can be shifted along the HOPG main axis direction *d*_1_ by multiples of two –CH_2_– units. Examples for this behavior are also seen in the packing faults that are indicated by arrows A–C in Figure 4e and f. In other words, slight changes of the backbone sizes can lead to a different interlocking of the side chains of adjacent macrocycles and thus may affect the packing of the macrocycles to an extent quite above the threshold of the experimental resolution.

Contrary to the different robustness of the tubular aggregates (gels) from these macrocycles (where the intraannular strands come into close contact) [22], no stability changes and no packing scheme changes for the 2D surface patterns are observed. Similar patterns with alike (porous polymorph) and only slightly varying (dense polymorph) unit cell parameters for all three compounds are formed. This is a clear result of the driving force for the pattern formation, which is – at least for the porous polymorph – a combination of van der Waals interactions between the molecules and the underlying graphite and the alkyl chain interdigitation between neighbored macrocycles. This shows that it is possible to vary the functionality of the macrocycle interior and to keep the pattern constant, which is of great relevance for the tailored design of functionalized adsorbate layers. Moreover, the distances of the macrocycles **1**–**3** (cf. Figure 5a) are alterable in a (more) predictable fashion along a specific direction (that shows the weakest intermolecular van der Waals interaction strength) as compared to the rather unspecific denser polymorphs of the hexagonal molecules described earlier (Figure 2). This can be drawn back to two discrete stabilities of different binding motifs that occur along the directions *d*_1_ as compared to *d*_2_ and *d*_3_.

## Conclusion

STM investigations of three shape-persistent macrocycles with different cavity fillings demonstrate that the ring interior has (rather) no effect on the 2D supramolecular surface patterns. Hence, this approach opens a way towards the tailored design of adsorbate layers that can carry functional groups in their interior. The pattern geometry is steered by the periphery and backbone of the macrocycle, whereas the layer functionalization is provided by the specific interior. Furthermore, the packings are closely related to the backbone structures and substitution patterns, for both, porous and dense polymorphs, and are alterable by compound concentration changes. The future investigations will include the role of size and flexibility of the intraannular strands on the supramolecular assembly in one and two dimensions, a strategy to attach functional units that point away from the surface to the third dimension in a pillar-like fashion, as well as the attachment of groups that selectively bind additional guest molecules that do not self-assemble without macrocycle template layers.

## Experimental

The synthesis and characterization of the compounds has been reported before [22]. STM was performed at the solution/solid interface under ambient conditions. 0.5 µL of a 10^−5^–2 × 10^−6^ M solution of the respective substance in 1,2,4-trichlorobenzene (TCB) was dropped onto a piece of freshly cleaved HOPG at elevated temperature (70 °C), and the sample was allowed to cool to rt prior to STM imaging. All STM measurements were performed in situ (with the tip immersed into the liquid) and typically completed within 30 min after the sample preparation. Bias voltages between −1.0 V and −1.2 V and current setpoints between 5 pA and 30 pA were applied to image the molecular adlayers shown in this work. Mechanically cut Pt/Ir (80:20) tips were used and further modified in situ by applying short voltage pulses. All STM images were in situ calibrated by subsequent immediate acquisition of an additional image at reduced bias voltage, therefore the atomic lattice of the HOPG surface is visible, which is used as a calibration grid. Data processing, also for image calibration, was performed using the SPIP 5 (Image Metrology) software package.
